# Toxicity of Cry- and Vip3Aa-Class Proteins and Their Interactions against *Spodoptera frugiperda* (Lepidoptera: Noctuidae)

**DOI:** 10.3390/toxins16040193

**Published:** 2024-04-16

**Authors:** Xiaobei Liu, Shen Liu, Shuxiong Bai, Kanglai He, Yongjun Zhang, Hui Dong, Tiantao Zhang, Zhenying Wang

**Affiliations:** 1 College of Plant Protection, Shenyang Agricultural University, Shenyang 110161, China; liuxiaobei402@163.com (X.L.); biocontrol@163.com (H.D.); 2 State Key Laboratory for the Biology of the Plant Diseases and Insect Pests, Institute of Plant Protection, Chinese Academy of Agricultural Sciences, Beijing 100193, China; liushen8152@163.com (S.L.); baishuxiong@caas.cn (S.B.); hekanglai@caas.cn (K.H.); yjzhang@ippcaas.cn (Y.Z.)

**Keywords:** *Bacillus thuringiensis*, bioassay, cry class, *Spodoptera frugiperda*, synergistic effect, Vip3Aa class

## Abstract

The fall armyworm (FAW), *Spodoptera frugiperda* (J.E. Smith), is one of the most important insect pests affecting corn crops worldwide. Although planting transgenic corn expressing *Bacillus thuringiensis* (Bt) toxins has been approved as being effective against FAW, its populations’ resistance to Bt crops has emerged in different locations around the world. Therefore, it is important to understand the interaction between different Bt proteins, thereby delaying the development of resistance. In this study, we performed diet-overlay bioassays to evaluate the toxicity of Cry1Ab, Cry1Ac, Cry1B, Cry1Ca, Cry1F, Cry2Aa, Cry2Ab, Vip3Aa11, Vip3Aa19, and Vip3Aa20, as well as the interaction between Cry1Ab-, Cry1F-, Cry2Ab-, and Vip3Aa-class proteins against FAW. According to our results, the LC_50_ values of Bt proteins varied from 12.62 ng/cm^2^ to >9000 ng/cm^2^ (protein/diet), among which the Vip3Aa class had the best insecticidal effect. The combination of Cry1Ab and Vip3Aa11 exhibited additive effects at a 5:1 ratio. Cry1F and Vip3Aa11 combinations exhibited additive effects at 1:1, 1:2, and 5:1 ratios. The combination of Cry1F and Vip3Aa19 showed an antagonistic effect when the ratio was 1:1 and an additive effect when the ratio was 1:2, 2:1, 1:5, and 5:1. Additionally, the combinations of Cry1F and Vip3Aa20 showed antagonistic effects at 1:2 and 5:1 ratios and additive effects at 1:1 and 2:1 ratios. In addition to the above combinations, which had additive or antagonistic effects, other combinations exhibited synergistic effects, with variations in synergistic factors (SFs). These results can be applied to the establishment of new pyramided transgenic crops with suitable candidates, providing a basis for FAW control and resistance management strategies.

## 1. Introduction

The fall armyworm (FAW), *Spodoptera frugiperda* (J. E. Smith) (Lepidoptera: Noctuidae), is native to tropical and subtropical regions of the Americas [1]. It was first discovered in China’s Yunnan province in December 2018 [2]. Since its invasion of China, it has become one of the major insect pests of corn, proliferating in corn cropping areas and causing significant losses to corn yield [3,4,5,6]. Currently, the control of FAW in China primarily relies on chemical insecticides. However, the increasing use of chemical insecticides has led to a series of environmental, socioeconomic, and pest resistance problems [5]. With the development of new biotechnology, transgenic crops expressing *Bacillus thuringiensis* (Bt) insecticidal protein not only effectively control the damage of target pests but also reduce the application of chemical insecticides, protect the environment, increase crop yield, and improve farmers’ income [7,8,9,10,11].

The commercial planting of transgenic Bt crops began in 1996, and the global planted area of Bt corn amounted to 60.9 million hectares in 2019, equivalent to 32% of the global planted area of corn [12]. However, large-scale planting of transgenic Bt crops has unintended consequences of targeting pests to undergo certain pressures, which may lead to increased resistance levels of target pests to Bt proteins [13,14,15,16,17,18,19,20,21,22,23,24]. Therefore, in order to avoid or delay the resistance evolution of target pests, the strategies of stacking two or more Bt insecticidal proteins in Bt plants have been deployed, with excellent results. Currently, Vip3Aa/Cry1Ac and Vip3Aa/Cry1Ac/Cry1Fa protein combinations have been used in cotton, and Vip3Aa/Cry1Ab and Vip3Aa/Cry1Ab/Cry1Fa have been used in corn to control the damage of target pests [25,26,27,28].

The Cry and Vip proteins produced by Bt have a similar mode of action, yet they do not compete for binding sites [29,30,31,32,33,34]. Some researchers have discovered that the combining of two or more Bt proteins is more effective against target pests compared to a single-Bt protein [35,36,37]. This combination not only provides a broader spectrum of insecticidal effects but also helps prevent or delay the evolution of resistance in target pests. However, it is important to note that the combination of different Bt proteins can exhibit synergistic or antagonistic effects. When two or more Bt proteins are mixed and fed to target pests, the insecticidal effect of the mixed proteins is lower than the expected insecticidal effect, which results in antagonism. The antagonism of target pests to a certain mixed-Bt protein will lead to the increase in the target pests’ resistance to the mixed-Bt protein or plants and eventually lead to the failure of the plant application of the mixed-Bt protein. Therefore, when combining different Bt proteins, we should consider the potential for synergistic or antagonistic effects. The combination of Bt proteins that do not compete for the same binding sites can achieve the purpose of avoiding or delaying the resistance evolution of target pests. However, the synergistic or antagonistic effects of the same Bt protein combination on different target pests may be different [38,39].

The single-Bt protein used in this study can be found in the *Bacillus thuringiensis* strain. At the same time, many new strains of B. thuringiensis have been sought that have a combination of toxin genes. For instance, the BTG strain contains seven genes encoding crystal toxins (cry1Ab35, cry1Db, cry1Fb, cry1Ib, cry2Ab, cry8Ea1, and cry9Ba) [40]; the ABTS-351 and YBT-1520 strains contain genes for Cry1Aa and Cry2Aa toxins; strain HD-29 contains plasmid-encoded cry1Aa, cry1Ac, cry1Ca, cry1Ia, cry9Ea, and vip3Aa [41]; and strain HD137 contains plasmid-encoded cry1Aa, cry1Ba, cry1Ca, and cry1Da [42]. Among them, cry1Ab, cry1F, vip3Aa, and other genes have been successfully applied in transgenic maize and have shown a good control effect on target pests. Therefore, these strains and proteins have great economic value in pest control. In this study, we have evaluated the efficacy of individual Cry and Vip3Aa proteins and their combinations against FAW, which can provide an important reference for the control of FAW and the establishment of new pyramided transgenic corn.

## 2. Results

### 2.1. Toxicity of Different Bt Proteins against S. frugiperda

For single proteins, the LC_50_ and LC_95_ values ranged from 12.62 ng/cm^2^ to >9000 ng/cm^2^ (protein/diet) and from 57.10 ng/cm^2^ to 6307.52 ng/cm^2^ (protein/diet), respectively (Table 1). The Cry1Ac protein showed less potency, with the highest LC_50_ value (>9000 ng/cm^2^), followed by the Cry1B protein (1750.04 ng/cm^2^), the Cry2Aa protein (688.02 ng/cm^2^), and the Cry1Ca protein (524.45 ng/cm^2^). The Cry1F, Cry2Ab, and Cry1Ab proteins also had good insecticidal activity, but the LC_50_ and LC_95_ values were lower than those of the Vip3Aa-class proteins. The LC_50_ of the Vip3Aa20 protein was 12.62 ng/cm^2^, which was not significantly different from that of Vip3Aa11 (12.93 ng/cm^2^) and Vip3Aa19 (16.52 ng/cm^2^).

### 2.2. Interactions between Cry-Class Proteins against S. frugiperda

The diet-overlay bioassay results of FAW with the Cry-class proteins in a 1:1 combination are shown in Table 2. Our results showed that the expected LC_50_ value of the Cry protein combination was significantly different from the observed LC_50_ value, with SF values ranging from 1.65 to 7.21, all of which were greater than 1, and exhibited a synergistic effect between these Cry protein combinations. Cry1Ab and Cry2Ab had the highest synergistic factor (SF = 7.21), and Cry1F and Cry2Ab had a lower synergistic factor (SF = 3.83). Cry1Ab and Cry1F had the smallest synergistic factor (SF = 1.65).

### 2.3. Interactions between Cry-Class and Vip3Aa-Class Proteins against S. frugiperda

The interactions between Cry1Ab- and Vip3Aa-class protein combinations at five ratios against FAW are shown in Table 3. The results showed that the combination of Cry1Ab and Vip3Aa11 at the ratio of 5:1 showed no significant difference between the expected value and the observed value, showing an additive effect. The expected values of all the other protein combinations were significantly greater than the observed values, and they all showed synergistic effects on larval control. The synergistic factor ranged from 1.58 (Cry1Ab + Vip3Aa11, ratio 1:5) to 3.50 (Cry1Ab + Vip3Aa19, ratio 5:1), a 2.2-fold difference between these protein combinations.

The interactions between Cry1F- and Vip3Aa-class protein combinations at several ratios against FAW are shown in Table 4. There was no significant difference between the expected value and the observed value at the ratios of 1:1, 1:2, and 5:1 for Cry1F and Vip3Aa11, but the expected value was significantly larger than the observed value at the ratios of 2:1 and 1:5, exhibiting a synergistic effect. When the ratio of Cry1F and Vip3Aa19 was 1:1, the expected value was significantly smaller than the observed value, showing an antagonistic effect. At other ratios, there was no significant difference between the expected value and the observed value. When the ratios of Cry1F and Vip3Aa20 were 1:1 and 2:1, there was no significant difference between the expected value and the observed value, but, at the ratio of 1:2 and 5:1, the expected values were significantly smaller than the observed value, exhibiting an antagonistic effect.

The interactions between Cry2Ab- and Vip3Aa-class protein combinations at several ratios against FAW are shown in Table 5. The results showed that the expected value of the combination of Cry2Ab- and Vip3Aa-class proteins was significantly higher than the observed value, indicating that all the protein combinations exhibited synergic action in the control of FAW larvae. The SF values of Cry2Ab- and Vip3Aa-class protein combinations ranged from 1.46 to 7.73, all of which were greater than 1, and they exhibited a synergistic effect. Cry2Ab and Vip3Aa19 had the highest synergistic factor at a 5:1 ratio (SF = 7.73), Cry2Ab and Vip3Aa11 had the lowest synergistic factor at 1:1 and 1:5 ratios (SF = 1.46), and the difference between these protein combinations was five-fold.

## 3. Discussion

*Spodoptera frugiperda* is an important agricultural pest that seriously threatens global food security and invaded China in 2018. Although transgenic corn has a good control effect on target pests, field-resistant populations have been detected in different areas due to long-term and large-scale planting of single-Bt corn [15,20,43,44]. As FAW is currently one of the most important lepidopteran pests globally, research into finding Bt proteins with commercial-level activity is needed. Therefore, in the present study, we evaluated the susceptibility of FAW to single Cry1-class, Cry2-class, and Vip3Aa-class proteins, as well as the susceptibility of FAW to the combination of Cry1Ab-, Cry1F-, Cry2Ab-, and Vip3Aa-class proteins, respectively.

Trypsin-activated Bt proteins were used in our study, as previous studies indicated that the conserved blocks, which are responsible for the toxic activity, located in the active toxic core of the protoxins would be released by the activation of midgut proteases [45]. Trypsin is one of the most important midgut proteases in lepidopteran larvae, which is responsible for Bt protein digestion [46]. The utilization of Trypsin-activated Bt proteins can better simulate the role of Bt proteins after entering the larval midgut.

The LC_50_ values of FAW to Cry-class and Vip3Aa-class proteins ranged from 12.62 ng/cm^2^ to >9000 ng/cm^2^ (protein/diet) (Table 1). Soares et al. [37] observed that Cry2Ab had a better insecticidal effect on FAW than Cry2Aa, which was consistent with the results observed in this study. Sena et al. observed that the LC_50_ values of FAW for Cry1Ab and Cry1F proteins were 867 ng/cm^2^ and 170 ng/cm^2^, respectively [30]; however, the LC_50_ values observed by us were lower than those observed by them. This may be related to factors such as the source and purification method of the protein. Moreover, Cry1Ca had a better insecticidal effect on FAW than Cry1Ab [37], but, in this study, Cry1Ab had a better insecticidal effect on FAW than Cry1Ca, which was different from previous results. Chinese researchers found that maize transgenic cry1Ab gene CM8101 and C0030.3.5 had a good control effect on FAW larvae [47,48]. Yang et al. found that Bt-Cry1Ab DBN9936 and Bt-Cry1Ab/Cry2Aj Ruifeng 125 maize could reduce the stress of 61.9~97.3% lepidopteran pests and avoid production losses of 16.4 to 21.3% (range from 11.9 to 99.2%) without the use of insecticides [49]. Transgenic Cry1Ab and Cry1F maize varieties have been cultivated in other countries for FAW control. However, FAW has developed resistance to Cry1Ab and Cry1F maize in the field through the long-term planting of single-Bt corn, but, so far, FAW has maintained a high sensitivity to Vip3Aa20 maize [13,20,50,51]. In a previous report, Vip3Aa proteins showed the best insecticidal effect on FAW, followed by Cry1F, Cry2Ab, and Cry1Ab [30,37,52,53], which is in agreement with our findings. Burkness et al. found that the average total number of larvae per ear of Bt11 corn was significantly higher than that of MIR162 corn, indicating that MIR162 corn had a better control effect on FAW than Bt11 corn [54]. Previous research results showed that the order of the lethal effect of Vip3A proteins on FAW was Vip3Af > Vip3Ae > Vip3Aa; these studies indicated that different Vip3A proteins had different lethal effects on FAW [39,55]. The Vip3Aa proteins selected in this study are Vip3Aa19 and Vip3Aa20, proteins which have been successfully expressed and commercially grown in cotton and corn plants. Previous studies have found that the LC_50_ value of the Vip3Aa protein of FAW ranges from 24.66 to 1650 ng/cm^2^ [30,37,38,39,55]. However, the LC_50_ values of the Vip3Aa proteins found by FAW were lower than their LC_50_ values, which may be related to factors such as the source of the protein and the purification method. Chakroun et al. found that the LC_50_ of FAW on the Vip3Aa protein was in the range of 4.4–15 ng/cm^2^ [56], which was comparable to the value measured by us.

Studies have shown that Cry1Fa is more toxic to FAW than Cry1Ab [57,58,59], suggesting that there are other limiting factors to the toxicity of Cry1Ab to FAW. The study found that a low toxicity of Cry1Ab was associated with reduced stability in the treatment of FAW midgut juice, decreased binding affinity to FAW BBMV, and decreased binding to FAW receptors (ALP and APN) [59,60]. It was also found that Cry1Ab was more sensitive to the midgut juice protease of FAW, while the higher stability of Cry1Fa to the FAW midgut juice protease could explain, in part, its greater toxicity, compared to Cry1Ab, for this insect pest [59,61]. In addition, studies have suggested that Cry1A and Cry1Fa toxins may depend on different receptor molecules [62]. The amino acid sequence similarity of Cry2Ab to Cry1Ab and Cry1Fa in domains II and III is 15% and 26%, with 22% and 31%, respectively [63]. Domains II and III, but not domain I, are involved in binding interactions that determine toxin specificity. Therefore, in this study, Cry1Ab, Cry1F, and Cry2Ab proteins with good insecticidal effects on FAW were selected and combined, respectively, to study their synergistic effects on FAW. Our study showed that the Cry1Ab + Cry2Ab combination exhibited a synergistic effect on FAW larvae, which was the same as that observed by Soares et al. [37]. Furthermore, we found synergistic effects of Cry1Ab + Cry1F and Cry2Ab + Cry1F on FAW larvae. In the Fatoretto report, Brazil approved Cry1F + Cry1Ab maize cultivation in 2011, but field failure started in 2012 [50]. Indeed, FAW had developed field resistance to Cry1F and Cry1Ab before planting Cry1F + Cry1Ab maize, which may have been the main reason for its planting failure. Synergistic or antagonistic effects were also found in some similar studies. After combination, Cry1Ac and Cry1Fa showed an additive interaction on *Helicoverpa armigera* (Hübner) (Lepidoptera: Noctuidae) and *Earias insulana* (Boisduval) (Lepidoptera: Noctuidae), whereas Cry1Ac and Cry2Ab interacted synergistically in mixtures comprising 1:1 or 1:4 of toxins against *H. armigera* and *E. insulana*. However, there was no synergism between Cry1Ac and Cry2Ab [64]. Su Mon et al. (2021) found that the combination Cry1Ab + Cry2Aa exhibited a slight-to-moderate antagonistic interaction on *Conogethes punctiferalis* (Guenée) (Lepidoptera: Crambidae) and slight synergism for Cry1Fa + Cry2Aa [36]. Although significant cross-resistance between Cry1 and Cry2 has been found in some pests [65,66,67,68,69,70,71,72], our results indicated that the combination of Cry1Ab, Cry1F, and Cry2Ab, in the ratio of 1:1, exhibited a synergistic effect on the control of FAW.

Vip3Aa-class proteins are highly toxic to FAW but not to *Ostrinia furnacalis* (Guenée) (Lepidoptera: Pyralidae) [73]. Cry1Ab has a good insecticidal effect against other target pests of corn, such as *C. punctiferalis*, *Mythimna separata* (Walker) (Lepidoptera: Noctuidae), and *H. armigera* [35,36]. We know that Vip3Aa proteins and Cry proteins have different mechanisms of action and do not compete for the same binding sites [29,30,33,37,74]. Therefore, the combination of Vip3Aa proteins and Cry proteins not only expand the insecticidal range but also delay the generation of resistance to target pests. Our study found that the combination of Cry1Ab and Vip3Aa11 had no significant difference between the expected value and the observed value at a ratio of 5:1. However, Cry1Ab and Vip3Aa proteins (Vip3Aa11, Vip3Aa19, and Vip3Aa20) showed significant differences in the expected values and the observed values at other ratios, exhibiting a synergistic effect. The combination of Cry2Ab and Vip3Aa proteins (Vip3Aa11, Vip3Aa19, and Vip3Aa20) showed significant differences in the expected value and the observed value at all ratios, all of which presented synergistic effects on FAW. Previous research results showed that Cry1Ab + Vip3Aa maize had a good control effect on FAW [48,54]. Wang et al. found that the Cry1Ab + Vip3Aa protein combination has a good effect on FAW [53]. Soares et al. [37] found that Cry1Ab or Cry2Ab proteins had synergistic effects on FAW when individually combined with Vip3Aa, which was consistent with the results observed in this study. The combinations of Cry1Ab or Cry1F proteins with the Vip3Aa19 protein both showed a synergistic effect on *C. punctiferalis* [36]. The combination of Cry1Ab or Cry1F with Vip3Aa16 showed a synergistic effect on M. separata [35]. The combination of Vip3Aa and Cry1Ia10 showed synergistic effects on FAW, *Spodoptera albula* (Lepidoptera: Noctuidae), and *Spodoptera cosmioides* (Lepidoptera: Noctuidae) larvae; however, it showed antagonism to the larvae of *Spodoptera eridania* (Lepidoptera: Noctuidae) [38]. These results indicated that the combination of Vip3Aa and Cry1 had different synergistic or antagonistic effects on different target pests. Therefore, local target pest species should be considered when using Bt protein combinations.

The above studies all adapted the method from Tabashnik to evaluate the interaction between Bt proteins; however, a relevant study used insect mortality to evaluate the interaction between Bt proteins [75]. In our study, the interactions of Bt protein combinations were evaluated first by the SF (method from Tabashnik), and then, to make our results more convincing, we conducted an ANOVA for 95%FLs of LC_50_. We considered a potential synergistic effect between Bt proteins if the ANOVA showed significance and the SF > 1. In the future, it will be necessary to use different methods for Bt protein interaction evaluations.

It is well known that Vip3Aa-class proteins and Cry1-class proteins do not compete for the same binding sites [34]. However, the mechanism behind their synergistic or antagonistic effects on target pests after combination remains unclear. Some researchers have speculated on the possibility that the proteins may physically interact with each other, isolating them from one another and forming a complex which makes both proteins inactive. Alternatively, the formation of the complex could just mask an epitope in the most toxic protein, preventing it from interacting with the membrane receptor. This antagonism may also arise from spatial interactions, where two toxins bind to different epitopes in the same molecular membrane [30,38,55,56,76,77,78,79,80,81,82,83]. Therefore, when planting transgenic plants with a Cry1 + Vip3Aa combination, we should consider the species of local target pests.

## 4. Conclusions

In our research, the combination of Cry1Ab + Vip3Aa11 (5:1 ratio), Cry1F + Vip3Aa11 (1:1, 1:2, and 5:1 ratios), Cry1F + Vip3Aa19 (1:2, 2:1, 1:5, and 5:1 ratios), and Cry1F + Vip3Aa20 (1:1 and 2:1 ratio) showed additive effects. The combination of Cry1F + Vip3Aa19 (1:1 ratio) and Cry1F + Vip3Aa20 (1:2 and 5:1 ratio) showed antagonistic effects. All other protein combinations showed synergistic effects, but the synergistic factors (SFs) differed. Therefore, planting transgenic plants with Cry1Ab + Vip3Aa or Cry2Ab + Vip3Aa can effectively control the damage of FAW, and the species of local target pests should also be considered. These results can be applied when establishing new pyramided transgenic crops with suitable candidates and serve as a basis for implementing FAW control and resistance management strategies. At the same time, it is critical to strengthen the ongoing monitoring of target pests and the adaptation of pest management strategies in response to evolving resistance dynamics.

## 5. Materials and Methods

### 5.1. Insects

The FAW original population was collected from the Yunnan province, China, in 2019, and its successive generations were feeding on an artificial diet (the main raw materials included soybean flour, wheat bran, yeast, sorbic acid, casein, ascorbic acid, mixed vitamins, and so on), without exposure to any Bt proteins. The study conducted by Li et al. (2019) revealed that the FAW that invaded China did not exhibit any resistance to Cry1Ab, Cry1Ac, Cry1F, Cry2Ab, and Vip3A proteins [52]. The population used in this experiment was collected in the fields of Yunnan in 2019 and was not exposed to any Bt preparations or proteins during indoor feeding. Therefore, it can be inferred that the FAW population used in this study did not develop any resistance to Bt proteins. The FAW population was reared at 26 ± 1 °C and 75% ± 5% relative humidity (RH) under a photoperiod of 16:8 h (L:D). Newly hatched neonate larvae (<24 h) were used for the diet bioassays.

### 5.2. Bt Proteins

Trypsin-activated Cry1Ab, Cry1Ac, Cry1Ca, Cry1B, Cry1F, and Cry2Aa proteins were purchased from Envirologix (Portland, OR, USA). Trypsin-activated Cry2Ab and Vip3Aa11 proteins were purchased from Meiyan Agricultural Technology Co., Ltd., Beijing, China. The Vip3Aa19 protein was purchased from Dabeinong Biotechnology Co., Ltd., Beijing, China. The Vip3Aa20 protein was provided by Syngenta Biotechnology Co., Ltd., Beijing, China. All Bt proteins were analyzed with SDS-PAGE gel to ensure their purity before use.

### 5.3. Bioassay

The insecticidal activity of Bt proteins against FAW neonate larvae was evaluated using concentration–response bioassays with the method of the surface of the artificial diet. Serial dilutions of eight or ten solutions (including a control) were prepared by decreasing the concentrations of the Bt protein with double-distilled water. Fifty microliters of the formulated solution were overlaid on the diet of a 24-well plate. After drying, single neonates (<24 h) were picked up with a fine brush and transferred to individual wells in 24-well plates. The plates were sealed with a membrane (Cat# 9733, Minnesota Mining and Manufacturing Company, Saint Paul, MN, USA) perforated with a sharp pin on each cell to provide aeration.

The toxicity of each Bt protein to FAW was determined separately. The combination of Cry1Ab + Cry2Ab, Cry1Ab + Cry1F, and Cry2Ab + Cry1F was mixed at a ratio of 1:1 to perform synergistic or antagonistic tests. For the Cry1Ab + Vip3Aa11, Cry1Ab + Vip3Aa19, Cry1Ab + Vip3Aa20, Cry1F + Vip3Aa11, Cry1F + Vip3Aa19, Cry1F + Vip3Aa20, Cry2Ab + Vip3Aa11, Cry2Ab + Vip3Aa19, and Cry2Ab + Vip3Aa20 combinations, more ratios (1:1, 1:2, 2:1, 1:5, and 5:1) were tested for the synergistic or antagonistic effect test. The type of interaction was evaluated using the formula of Tabashnik [84]. For more details on the concentrations used, see Supplementary Materials Table S1.

For each concentration of Bt protein (single/combination), 24 neonate larvae were used as a replicate and 3 replicates were performed for each concentration (a total of 72 larvae per concentration). The number of dead larvae was recorded after 7 days, and the acceptable control mortality rate was less than 20%. The criteria for determining the death of larvae in the treatment group (Bt protein added) and the control group (Bt protein-free) were as follows: the larvae were counted as dead if they did not move when touching the tail with a brush or they did not growth to the second instar (L_2_ stage) [85]. All the bioassay larvae were maintained in the rearing room at 26 ± 1 °C and 75% ± 5% relative humidity (RH) under a photoperiod of 16:8 h (L:D).

### 5.4. Statistical Analysis

The mortality of each treatment (including single-protein treatments and mixtures of proteins) was analyzed by probit regression using PoloPlus (LeOra Software, Version 1.0), which generated LC_50_ values with 95% fiducial limits (FLs), chi-square (χ^2^), slope with standard errors (slope ± SE), and degrees of freedom (df). The expected LC_50_ values and synergistic factor (SF) were evaluated using the formula from Finney, transformed by Tabashnik [84]:

$${\mathrm{LC}}_{50}\;(m)=\frac{1}{\frac{r_{a}}{{\mathrm{LC}}_{50}\;(a)\;}+\frac{r_{b}}{{\mathrm{LC}}_{50}\;(b)\;}}$$

where LC_50_ (m) is the expected LC_50_ of the mixture, LC_50_ (a) and LC_50_ (b) are the respective LC_50_ of protein a and protein b, and r_a_ and r_b_ are the relative proportions of proteins a and b in the mixture, respectively. The same formula was used to determine the 95% FLs. The synergistic factor (SF) of the protein combinations was determined by dividing the expected LC_50_ by the observed LC_50_ values. A value of SF > 1 indicated a synergistic interaction, SF (<1) indicated an antagonistic interaction, and SF = 1 indicated additive toxicity [86,87]. The synergism was judged not only by whether the SF value was greater than 1 but also by whether there existed a significant difference between the expected value and the observed value.

## Figures and Tables

**Table 1:** Toxicity of different Bt proteins against *Spodoptera frugiperda* neonate larvae.

Bt Proteins	n	LC_50_ (95% FLs) ng/cm^2 A^	LC_95_ (95% FLs) ng/cm^2 A^	Slope ± SE	χ^2^	df
Cry1Ab	576	271.19 (212.91–346.66) c	6307.52 (3708.61–13,210.67) a	1.20 ± 0.11	8.15	22
Cry1Ac	432	>9000	-	1.21 ± 0.16	14.03	16
Cry1B	504	1750.04 (1373.41–2203.72) a	-	1.26 ± 0.10	18.54	19
Cry1Ca	432	524.45 (308.03–788.56) b	-	0.79 ± 0.11	10.32	16
Cry1F	576	19.50 (15.06–24.79) e	238.91 (170.97–361.11) c	1.51 ± 0.11	12.10	22
Cry2Aa	384	688.02 (542.58–857.12) b	-	1.46 ± 0.14	11.89	17
Cry2Ab	480	72.01 (52.18–93.08) d	943.51 (655.76–1569.23) b	1.47 ± 0.15	15.17	18
Vip3Aa11	408	12.93 (10.72–15.15) f	57.10 (45.19–79.09) e	2.55 ± 0.26	4.54	15
Vip3Aa19	408	16.52 (11.89–21.12) ef	91.91 (66.68–150.88) de	2.21 ± 0.27	15.99	15
Vip3Aa20	504	12.62 (10.13–15.19) f	92.80 (69.70–137.15) d	1.90 ± 0.18	12.17	19

n: number of larvae in the probit analysis. 95% FLs: 95% fiducial limits. ^A^: values followed by the same lowercase letter in the same column indicate no significant difference (overlapping 95% fiducial limits). SE: standard error. χ^2^: chi-square. df: degrees of freedom.

**Table 2:** Susceptibility of *Spodoptera frugiperda* neonate larvae to combinations of Cry-class proteins at a ratio 1:1.

Bt Proteins	Ratio	N	Observed LC_50_ (95% FLs) ng/cm^2 B^	Expected LC_50_ (95% FLs) ng/cm^2 B^	SF	Slope ± SE	χ^2^	df
Cry1Ab + Cry1F	1:1	576	22.07 (16.73–28.17) b	36.38 (28.13–46.27) a	1.65	1.39 ± 0.14	14.04	22
Cry1Ab + Cry2Ab	1:1	576	15.78 (12.59–19.36) b	113.80 (83.82–146.76) a	7.21	1.48 ± 0.12	6.04	22
Cry1F + Cry2Ab	1:1	576	8.01 (6.33–9.93) b	30.69 (23.37–39.15) a	3.83	1.39 ± 0.11	8.96	22

n: number of larvae in the probit analysis. 95% FLs: 95% fiducial limits. Expected LC_50_ ng/cm^2^: calculated according to Tabashnik’s formula. ^B^: values followed by the same lowercase letter in the same row indicate no significant difference (overlapping 95% fiducial limits). SF: synergistic factor, the excepted LC_50_ value divided by the observed LC_50_ value. SE: standard error. χ^2^: chi-square. df: degree of freedom.

**Table 3:** Susceptibility of *Spodoptera frugiperda* neonate larvae to Cry1Ab- and Vip3Aa-class proteins at different ratios.

Bt Proteins	Ratio	n	Observed LC_50_ (95% FLs) ng/cm^2 B^	Expected LC_50_ (95% FLs) ng/cm^2 B^	SF	Slope ± SE	χ^2^	df
Cry1Ab + Vip3Aa11	1:1	504	8.85 (6.90–10.91) b	24.68 (20.41–29.03) a	2.79	1.81 ± 0.16	8.23	19
	1:2	384	9.28 (7.37–11.41) b	18.94 (15.69–22.24) a	2.04	2.67 ± 0.26	8.10	14
	2:1	480	14.85 (12.57–17.47) b	35.41 (29.22–41.80) a	2.38	2.06 ± 0.16	6.24	18
	1:5	504	9.75 (8.29–11.45) b	15.37 (12.74–18.02) a	1.58	1.96 ± 0.14	12.27	19
	5:1	432	50.52 (29.17–63.35) a	62.65 (51.38–74.60) a	1.24	3.13 ± 0.77	8.60	16
Cry1Ab + Vip3Aa19	1:1	504	10.63 (8.05–13.41) b	31.14 (22.52–39.81) a	2.93	1.62 ± 0.17	15.16	19
	1:2	576	10.74 (8.77–12.99) b	24.05 (17.35–30.74) a	2.24	1.63 ± 0.13	10.14	22
	2:1	576	24.09 (19.82–28.98) b	44.18 (32.09–56.48) a	1.83	1.69 ± 0.13	9.15	22
	1:5	576	8.68 (6.98–10.69) b	19.59 (14.11–25.04) a	2.26	1.67 ± 0.14	23.87	22
	5:1	504	21.73 (18.15–25.61) b	75.98 (55.77–97.13) a	3.50	2.17 ± 0.19	15.62	19
Cry1Ab + Vip3Aa20	1:1	456	10.37 (8.55–12.37) b	24.12 (19.34–29.10) a	2.33	2.29 ± 0.22	9.05	17
	1:2	408	9.37 (7.90–10.97) b	18.5 (14.84–22.30) a	1.97	2.57 ± 0.23	13.61	15
	2:1	384	13.37 (11.27–15.82) b	34.64 (27.75–41.90) a	2.59	2.16 ± 0.18	7.58	14
	1:5	504	5.80 (4.88–6.80) b	15.00 (12.04–18.07) a	2.59	2.25 ± 0.19	13.18	19
	5:1	456	26.54 (21.92–31.56) b	61.43 (49.10–74.76) a	2.31	2.63 ± 0.26	10.85	17

n: number of larvae in the probit analysis. 95% FLs: 95% fiducial limits. Expected LC_50_ ng/cm^2^: calculated according to Tabashnik’s formula. ^B^: values followed by the same lowercase letter in the same row indicate no significant difference (overlapping 95% fiducial limits). SF: synergistic factor, the excepted LC_50_ value divided by the observed LC_50_ value. SE: standard error. χ^2^: chi-square. df: degree of freedom.

**Table 4:** Susceptibility of *Spodoptera frugiperda* neonate larvae to Cry1F- and Vip3Aa-class proteins at different ratios.

Bt Proteins	Ratio	n	Observed LC_50_ (95% FLs) ng/cm^2 B^	Expected LC_50_ (95% FLs) ng/cm^2 B^	SF	Slope ± SE	χ^2^	df
Cry1F + Vip3Aa11	1:1	504	14.73 (12.58–17.07) a	15.55 (12.52–18.81) a	1.06	2.79 ± 0.26	11.92	19
	1:2	432	13.10 (11.03–15.46) a	14.57 (11.86–17.41) a	1.11	2.22 ± 0.17	9.20	16
	2:1	576	12.44 (10.70–14.52) b	16.68 (13.27–20.45) a	1.34	2.11 ± 0.15	10.70	22
	1:5	576	8.90 (7.51–10.40) b	13.70 (11.26–16.20) a	1.54	2.37 ± 0.21	8.12	22
	5:1	648	15.08 (12.91–17.66) a	17.98 (14.11–22.41) a	1.19	1.98 ± 0.13	10.99	25
Cry1F + Vip3Aa19	1:1	528	47.36 (41.58–52.96) a	17.89 (13.29–22.81) b	0.38	5.07 ± 0.68	8.39	20
	1:2	480	19.82 (14.80–25.55) a	17.41 (12.79–22.22) a	0.88	1.76 ± 0.16	25.88	18
	2:1	504	30.44 (21.39–44.72) a	18.39 (13.83–23.43) a	0.60	1.20 ± 0.10	35.36	19
	1:5	504	14.77 (11.85–18.26) a	16.95 (12.32–21.65) a	1.15	1.75 ± 0.13	21.76	19
	5:1	576	12.28 (9.71–15.33) a	18.93 (14.42–24.09) a	1.54	1.29 ± 0.11	11.79	22
Cry1F + Vip3Aa20	1:1	576	13.48 (11.55–15.60) a	15.32 (12.11–18.84) a	1.14	2.64 ± 0.24	12.13	22
	1:2	648	21.31 (18.35–24.34) a	14.30 (11.37–17.44) b	0.67	3.52 ± 0.39	6.70	22
	2:1	552	14.46 (12.25–17.06) a	16.50 (12.96–20.48) a	1.14	1.94 ± 0.13	6.06	21
	1:5	552	8.90 (7.45–10.48) b	13.41 (10.71–16.24) a	1.51	2.32 ± 0.20	9.17	21
	5:1	624	29.36 (23.91–35.71) a	17.88 (13.93–22.43) b	0.61	1.75 ± 0.16	13.47	24

n: number of larvae in the probit analysis. 95% FLs: 95% fiducial limits. Expected LC_50_ ng/cm^2^: calculated according to Tabashnik’s formula. ^B^: values followed by the same lowercase letter in the same row indicate no significant difference (overlapping 95% fiducial limits). SF: synergistic factor, the excepted LC_50_ value divided by the observed LC_50_ value. SE: standard error. χ^2^: chi-square. df: degree of freedom.

**Table 5:** Susceptibility of *Spodoptera frugiperda* neonate larvae to Cry2Ab- and Vip3Aa-class proteins at different ratios.

Bt Proteins	Ratio	n	Observed LC_50_ (95% FLs) ng/cm^2 B^	Expected LC_50_ (95% FLs) ng/cm^2 B^	SF	Slope ± SE	χ^2^	df
Cry2Ab + Vip3Aa11	1:1	504	15.02 (11.99–18.12) b	21.92 (17.79–26.06) a	1.46	2.97 ± 0.35	22.55	19
	1:2	480	10.59 (8.28–12.88) b	17.80 (14.58–21.01) a	1.68	2.56 ± 0.29	15.54	18
	2:1	480	11.71 (9.54–13.87) b	28.54 (22.79–34.29) a	2.44	2.80 ± 0.32	11.00	18
	1:5	456	10.24 (8.47–12.19) b	14.98 (12.36–17.61) a	1.46	2.32 ± 0.22	7.41	17
	5:1	504	13.88 (11.60–16.29) b	40.88 (31.73–50.12) a	2.95	2.55 ± 0.26	8.84	19
Cry2Ab + Vip3Aa19	1:1	648	10.95 (8.89–13.24) b	26.87 (19.37–34.43) a	2.45	1.81 ± 0.15	7.91	25
	1:2	552	6.44 (5.06–8.06) b	22.23 (16.01–28.45) a	3.45	1.31 ± 0.11	14.98	21
	2:1	552	7.21 (5.56–9.15) b	33.97 (24.50–43.58) a	4.71	1.31 ± 0.11	14.82	21
	1:5	552	7.63 (6.03–9.60) b	18.95 (13.65–24.24) a	2.48	1.41 ± 0.11	8.56	21
	5:1	348	5.97 (4.22–8.07) b	46.17 (33.35–59.37) a	7.73	1.36 ± 0.15	13.41	14
Cry2Ab + Vip3Aa20	1:1	384	6.57 (4.97–8.33) b	21.48 (16.97–26.12) a	3.27	1.95 ± 0.19	8.65	14
	1:2	408	7.45 (5.33–9.43) b	17.40 (13.85–21.07) a	2.34	2.59 ± 0.38	7.57	15
	2:1	552	9.04 (7.47–10.78) b	28.03 (21.89–34.36) a	3.10	2.05 ± 0.18	9.09	21
	1:5	432	4.60 (3.54–5.65) b	14.63 (11.70–17.65) a	3.18	2.51 ± 0.31	7.10	16
	5:1	384	9.41 (7.61–11.33) b	40.36 (30.84–50.19) a	4.29	2.30 ± 0.22	8.59	14

n: number of larvae in the probit analysis. 95% FLs: 95% fiducial limits. Expected LC_50_ ng/cm^2^: calculated according to Tabashnik’s formula. ^B^: values followed by the same lowercase letter in the same row indicate no significant difference (overlapping 95% fiducial limits). SF: synergistic factor, the excepted LC_50_ value divided by the observed LC_50_ value. SE: standard error. χ^2^: chi-square. df: degree of freedom.

## Data Availability

Data are contained within the article and supplementary materials.

## Supplementary Materials

The following supporting information can be downloaded at https://www.mdpi.com/article/10.3390/toxins16040193/s1: Figure S1: Bt proteins; Table S1: Bioassay of Bt protein concentration.
