# Supplementary material for: Toxicity of Cry- and Vip3Aa-Class Proteins and Their Interactions against Spodoptera frugiperda (Lepidoptera: Noctuidae)
Source: Toxins (Basel). 2024 Apr 16;16(4):193. doi: 10.3390/toxins16040193 (PMC11053954; doi:10.3390/toxins16040193)
Supplement: Supplementary file 1 [file toxins-16-00193-s001.zip › Figure S1.pdf]

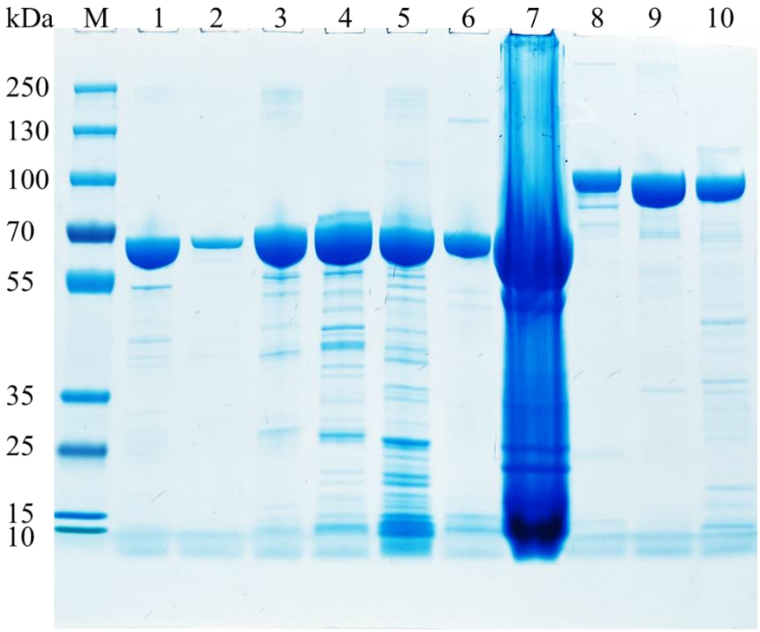

Figure S1. Bt proteins.

Notes: M: marker; 1: Cry1Ab; 2: Cry1Ac; 3: Cry1B; 4: Cry1Ca; 5: Cry2Aa; 6: Cry2Ab; 7: Cry1F; 8: Vip3Aa11; 9: Vip3Aa19; 10: Vip3Aa20. All the Bt proteins are trypsin-activated.
